# Role of salvage lymph node dissection in patients previously treated for prostate cancer: systematic review

**DOI:** 10.1590/S1677-5538.IBJU.2020.0051

**Published:** 2020-08-20

**Authors:** João Paulo Pretti Fantin, Maria Claudia Bicudo Furst, Marcos Tobias-Machado, Roberto Lodeiro Muller, Roberto Dias Machado, Alexandre Cesar Santos, Wesley Justino Magnabosco, Cinthia Alcantara-Quispe, Eliney Ferreira Faria

**Affiliations:** 1 Hospital de Câncer de Barretos Hospital do Amor BarretosSP Brasil Hospital de Câncer de Barretos - Hospital do Amor, Barretos, SP, Brasil; 2 Faculdade de Medicina do ABC Departamento de Urologia Santo AndréSP Brasil Departamento de Urologia, Faculdade de Medicina do ABC, Santo André, SP, Brasil

**Keywords:** Prostate cance, familial [Supplementary Concept], Lymph Nodes, Systematic Reviews as Topic

## Abstract

Prostate cancer is the most common invasive cancer in men. Radical prostatectomy (RP) is a definitive treatment option, but biochemical recurrence can reach 40%. Salvage lymphadenectomy is a relatively recent approach to oligometasis and has been rapidly diffused primarily due to improvement in imaging diagnosis and results showing possibly promising therapy. A systematic literature review was performed in March 2020, according to the PRISMA statement. We excluded studies with patients with suspicion or confirmation of visceral and / or bone metastases. A total of 27 articles were included in the study. All studies evaluated were single arm, and there were no randomized studies in the literature. A total of 1,714 patients received salvage lymphadenectomy after previous treatment for localized prostate cancer. RP was the most used initial therapeutic approach, and relapses were based on PET / CT diagnosis, with Coline-11C being the most widely used radiopharmaceutical. Biochemical response rates ranged from 0% to 80%. The 5 years - Free Survival Biochemical recurrence was analyzed in 16 studies with rates of 0% up to 56.1%. The articles do not present high levels of evidence to draw strong conclusions. However, even if significant rates of biochemical recurrence are not evident in all studies, therapy directed to lymph node metastases may present good oncological results and postpone the onset of systemic therapy. The long-term impact in overall survival and quality of life, as well as the best strategies for case selection remains to be determined.

## INTRODUCTION

Prostate cancer (PCa) is the most common invasive cancer in men (1). Despite more accurate case selection using modern imaging techniques and advances in treatment (either surgery or radiation therapy) (2), recurrence after primary curative treatment remains significant. Overall, studies have found biochemical recurrence (BR) and clinical recurrence rates of approximately 30-40% (2-5) and 15% (6, 7), respectively.

Typically, the pattern of PSA rise after primary treatment suggests if recurrence is in the prostatic fossa or extraprostatic. Patients with biochemical recurrence are usually referred to radiation therapy if residual disease in prostatic fossa is suspected (8) or to androgen deprivation therapy (ADT) if lymph node or systemic metastasis are detected (9, 10). Recently, refinements in imaging methods, such as magnetic resonance imaging (MRI), computed tomography (CT), ^11^C-choline positron emission tomography (PET)/CT (11-16) and more recently, 68-Gadolinium-prostate-specific membrane antigen (PSMA) (17-21), have allowed clinicians to distinguish between patients with lymph node recurrence and others with bone or visceral metastasis (22).

Interestingly, patterns of clinical recurrence are associated with distinct outcomes. Indeed, accumulating evidence indicates patients with oligometastasis have better prognosis after ADT compared to extensive metastasis, with a median survival of over 6 years (23). In addition of the number of metastatic foci impacting on outcomes, a recent study on the natural history of patients with exclusive lymph nodes metastasis (one of the most common sites of metastasis) (24), reported better prognosis among those patients compared to patients with bone and/or visceral metastases (4). Another evidence suggesting patients with exclusive lymph node metastasis may achieve better prognosis arises from occasional sustained PSA relapse-free rates among intermediate- and high-risk patients with limited nodal disease when complete resection is obtained during primary treatment (6, 25-28). Thus, patients with exclusive lymph node recurrence may be good candidates for salvage treatment with metastasis-directed therapy (MDT), resulting in complete PSA responses among a significant number of cases (9, 10, 13, 29-37). Those patients would certainly benefit of having ADT receipt (and its side-effects) spared or postponed.

Nevertheless, evidence favoring MDT for recurrence after primary treatment for PCa is still unclear. Prior studies on this subject are very heterogeneous, with relatively small sample size and wide variation in PSA response. Recently, Ploussard et al. (38) showed a systematic review showing heterogenous studies. In our study, we assessed with a systematic review studies reporting the outcomes SLND for MDT among patients treated for localized PCa who had recurrence with exclusive lymph node oligometastases.

## MATERIAL AND METHODS

We aimed to include in this systematic review studies reporting on patients with PCa treated with curative intent and recurrence pattern of exclusive lymph node metastasis. Patients subsequentially underwent MDT with SLND and in some cases with additional radiation therapy and ADT. Based on these overall studies characteristics, MEDLINE, EMBASE, Cochrane, and Web of Science databases were queried using appropriate MEdical Subject Headings (MESH) terms to retrieve publications in English language. We scrutinized results from main query in stepwise approach, initially reviewing title and structured abstracts, and posteriorly with thorough assessment of full text by two independent researchers. Occasional lack of consensus was resolved by another ad hoc reviewer. In addition, articles deemed of interest could be manually included based on the examination of the references sections of retrieved studies from primary query. There was no filtering on past publication date.

We excluded studies including patients with visceral and/or bone metastases. Oncological endpoints of interest were specific cancer survival (SCS), biochemical relapse-free survival (BRFS) and clinical relapse-free survival (CRFS).

We followed the guidelines of the Preferred Reporting Items for Systematic Reviews and Meta-Analyses (PRISMA) protocol (39). Methodological quality for identifying limitations among studies was assessed by the Scottish Intercollegiate Guidelines Network (SIGN) checklist. Cohort and case-control studies were scored using SIGN checklist #3 and #4, respectively.

### Statistical Analysis

Data were assessed on the basis of the oncological outcomes mentioned above. The continuous variables were assessed using the mean difference, which adopts a 95% confidence interval.

## RESULTS

A total of 2.936 articles were retrieved from intended databases queries (MEDLINE: 956, EMBASE: 1.366, Cochrane: 71, and Web of Science: 543). No additional study was manually selected. The searches were concluded in March 25^th^, 2020.

**Figure 1:**
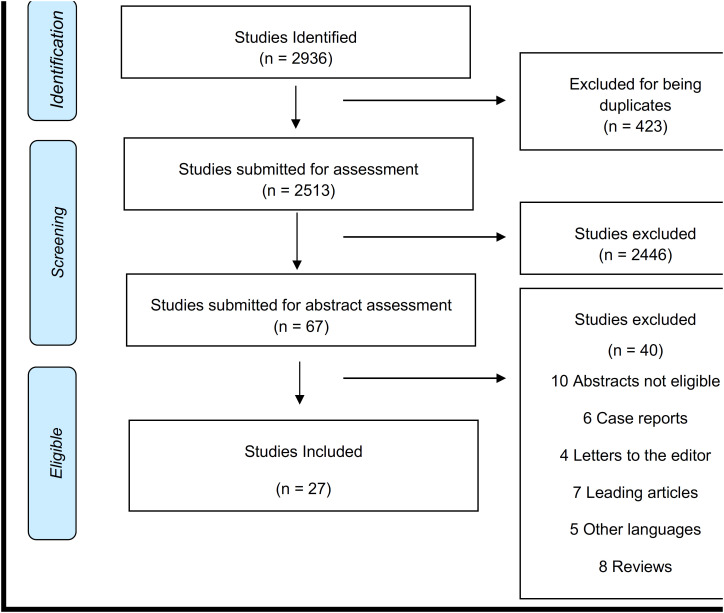
Flowchart (Preferred Reporting Items foflow diagram).

Figure-1 shows the flowchart of the selection of the articles.

After assessment by reviewers, 27 studies were selected for analysis. Due to heterogeneous studies approaches, unpaired data, and absence of comparative analysis among them, a systematic review solely of the descriptive analyses was performed. Studies characteristics and methodologies are detailed in Table-1. Publication dates among selected studies ranged from 2008 to 2020. In all studies, Radical Prostatectomy (RP) was the most common primary treatment, followed by radiation therapy, which was reported in eight studies; two studies reported the use of high-intensity focused ultrasound, despite limited to few patients. Ten studies were prospective and 17 retrospectives. None of them were randomized clinical trials.

**Table 1:** Descriptive and oncological data from the selected studies.

Author	Initial treatment	Approach type	Study type	Patient number	Age (median)	Diagnosis (PET/CT)	PSA level on relapse	Positive histological findings	Complete Response	Follow up (median) months	Lymph nodes removed (median)	Positive lymph node findings (median)	SCS (5 years)	BRFS (5 years)	CRFS (5 years)
**Schilling et al. 2008** (46)	RP / RT	VLP	Retrospective	10	n/r	^11^C-Choline	10,8	70%	**n/r**	11	7	**n/r**	**n/r**	**n/r**	**n/r**
**Rinnab et al. 2008** (47)	RP	Open	Prospective	15	62,1	^11^C-Choline	2,34	53,33%	6,67%	13,7	13,9	**n/r**	**n/r**	**n/r**	**n/r**
**Winter et al. 2010** (48)	RP +LDN	Open	Prospective	6	61,7	^11^C-Choline	2,38	100,00%	50,00%	24	10	**n/r**	**n/r**	**n/r**	**n/r**
**Rigatti et al. 2011** (31)	RP	Open	Prospective	72	66,9	^11^C-Choline	3,7	83,33%	56,94%	39,8	30,6	2	75%	19%	34%
**Jilg et al. 2012** (29)	RP / RT	Open	Prospective	52	65	^11^C-Choline / ^18^F-Choline	11,1	90,38%	46,15%	35,5	17	4	77,70%	8,70%	25,60%
**Martini et al. 2012** (16)	RP	Open	Retrospective	8	n/r	^11^C-Choline	1,62	75,00%	12,50%	29	11,5	**n/r**	**n/r**	**n/r**	**n/r**
**Osmonov et al. 2014** (49)	RP	Open	Retrospective	41	66,1	^11^C-Choline	n/r	**n/r**	**n/r**	24	16,6	**n/r**	100%	56,1%	**n/r**
**Passoni et al. 2014** (13)	RP	Open	Prospective	46	61	^11^C-Choline	0,5	65,22%	**n/r**	**n/r**	13	**n/r**	**n/r**	**n/r**	**n/r**
**Suardi et al. 2015** (32)	RP	Open	Prospective	59	64	^11^C-Choline	3,95	79,66%	59,32%	76,6	26	**n/r**	89,1%	29,40%	52%
**Rischke et al. 2015** (51)	RP/RT	Open	Retrospective	46	65	^11^C-Choline / ^18^F-Choline	3,5	100,00%	65,22%	28,7	25,5	**n/r**	77,70%	26,30%	**n/r**
**Claeys et al. 2015** (53)	RP/RT/HIFU	Open / Robotic/VLP	Prospective	17	65	^11^C-Choline / ^18^F-Choline	2,01	88,24%	17,65%	22	11	**n/r**	**n/r**	**n/r**	**n/r**
**Karnes et al. 2015** (9)	RP	Open	Retrospective	52	60	^11^C-Choline	2,2	**n/r**	57,69%	20	21,5	3,5	92,50%	45,50%	46,90%
**Tilki et al. 2015** (33)	RP	Open	Retrospective	58	n/r	^18^F-Choline	9,8	77,59%	22,41%	39	18,6	**n/r**	71,10%	0%	35,90%
**Winter et al. 2015** (15)	RP+LDN	Open	Prospective	13	61	^11^C-Choline / ^18^F-Choline	1,64	84,62%	38,46%	72	**n/r**	**n/r**	**n/r**	**n/r**	**n/r**
**Hijazi et al. 2015** (17)	RP	Open / Robotic	Retrospective	17	71	^68^Ga PSMA	2,9	**n/r**	82,35%	3	10	**n/r**	**n/r**	**n/r**	**n/r**
**Osmonov et al. 2016** (50)	RP/ RP+LDN/RT	Open	Retrospective	54	n/r	^11^C-Choline	6,7	**n/r**	40,74%	42,7	20	4,1	80,60%	40%	**n/r**
**Zattoni et al. 2016** (35)	RP	Open	Retrospective	117	65	^11^C-Choline	2,3	99,15%	79,49%	20,2	22	**n/r**	97%	31%	51%
**Porres et al. 2017** (52)	RP/RT/HIFU	Open	Retrospective	87	66,7	^18^F-Choline / ^68^Ga PSMA	2,63	21,84%	70,11%	21	12	1	89,8%*	27,3%*	62,5%*
**Montorsi et al. 2017** (54)	RP/PTR+LDN	Robotic	Retrospective	16	66	^68^Ga PSMA / ^18^F-Choline	1	68,75%	31,25%	12	16,5	4	**n/r**	**n/r**	**n/r**
**Siriwardana et al. 2017** (20)	RP/ RP+LDN/RT	Robotic	Retrospective	35	67	^68^Ga PSMA	2,2	91,43%	42,86%	12	9	2	**n/r**	23%	66%
**Abreu et al. 2017** (55)	RP/RT	Robotic	Prospective	10	65	^11^C-Choline	2,78	70,00%	0,00%	**n/r**	83	8	**n/r**	**n/r**	**n/r**
**Herlemann et al. 2017** (21)	RP	Open	Prospective	104	64	^68^Ga PSMA / ^18^F-Choline	4,1	82,69%	29,81%	39,5	13	3	82,8	6,20%	26,00%
**Otta-Oshiro et al. 2019** (36)	RP/RT	Open/VLP	Retrospective	23	67	^11^C-Choline	4,63	**n/r**	13%	82	19	6	**n/r**	**n/r**	**n/r**
**Linxweiler et al. 2018** (56)	RP/RP+LDN	Robotic	Retrospective	36	66	^68^Ga PSMA / ^18^F-Choline	1,98	**n/r**	36%	30	6,5	1	**n/r**	23%**	66%**
**Fossati et al. 2019** (57)	RP/RP+RT	Open/VLP/Robotic	Retrospective	654	66	^68^Ga PSMA / ^11^C-Choline	2,1	**n/r**	44%	30	26	**n/r**	**n/r**	25%*	55%*
**Hanske et al. 2019** (37)	RP	Open	Retrospective	43	62	^68^Ga PSMA	0,8	69,47%	18,6%	22	21	1	**n/r**	6,98%**	**n/r**
**Boeri et al. 2020** (43)	RP	Open	Retrospective	23	n/r	^11^C-Choline	2,5	**n/r**	69,6%	49,3	19	4	72,7%	19,3%*	**n/r**

**RP** = radical prostatectomy; **RT** = radiotherapy; **LDN** = lymphadenectomy; **VLP** = video laparoscopy; **PSMA** = prostate-specific membrane antigen; **n/r** = not reported; **SCS** = specific cancer survival; **BRFS** = biochemical relapse-free survival; **CRFS** = clinical relapse-free survival;

* period of 3 years;

** period of 1 year.

Among selected studies, a total of 1.714 patients with median age of 65 years received SLND for MDT after recurrence. Median PSA level found prior to SLND was 3.58ng/mL. All studies used PET/CT for detection of metastasis, and radionuclides employed varied among studies; most of them used 11C-Choline PET/CT (40), in addition to 18F-Choline, 18F-FDG and 11C-Acetate. Winter et al. (15) introduced in 2015 68Gadolinium-PSMA PET/CT, a new radionuclide for detecting lymph node recurrence.

In Table-1, cancer outcomes are shown. Of 27 studies assessed, 24 studies assessed serum PSA response after SLND. A PSA level of <0.2ng/mL 40 days after the procedure was considered complete response. With that criterion, studies had striking diversity of complete PSA response rates, ranging from 0 to >80%. Among 16 studies reporting 5-year BRFS, results varied from 0 to 56.1%.

Regarding safety of SLND, 20 studies reported complications using Clavien-Dindo classification system (41). Clinically significant complications (Grade 3 or above) occurred among 1.69% and 30% of patients who underwent SLND, depending on the study. No study reported a life-threatening complication; seven studies presented no results on safety of SLND.

Methodological quality of cohort studies is shown on Table-2, where methodological quality was considered low in 19 studies and acceptable in other 6 studies. Two case-control studies had acceptable quality using SIGN checklist. No study had high methodological quality.

**Table 2:** Scottish Intercollegiate Guidelines Network, Methodology Ckecklist 3: Cohort Studies, Version 3.0.

Methodology Ckecklist 3: Cohort Studies	Section 1: Internal Validity														Section 2: Overall assessment of the study			
	Clearly focused question	Selection of subjects					Assessment						Confounding	Statistical Analysis				
	1.1	1.2	1.3	1.4	1.5	1.6	1.7	1.8	1.9	1.10	1.11	1.12	1.13	1.14	2.1	2.2	2.3	2.4
**Schilling et al. 2008** (46)	No	Not apply	Not apply	Not apply	0	Not apply	No	Not apply	No	No	No	No	No	No	Low Quality	No	No	
**Rinnab et al. 2008** (47)	Yes	Not apply	Not apply	Not apply	0	Not apply	Yes	Not apply	No	Yes	No	No	No	No	Low Quality	No	No	
**Winter et al. 2010** (48)	Yes	Not apply	Not apply	Not apply	0	Not apply	No	Not apply	No	Yes	No	No	No	No	Low Quality	No	No	
**Rigatti et al. 2011** (31)	Yes	Not apply	Not apply	Not apply	0	Not apply	Yes	Not apply	No	Yes	Yes	No	Yes	Yes	Acceptable	No	No	
**Jilg et al. 2012** (29)	Yes	No	Not apply	Not apply	0	Not apply	Yes	No	No	Yes	No	No	Yes	Yes	Low Quality	No	No	
**Martini et al. 2012** (16)	No	Not apply	Not apply	Not apply	0	Not apply	No	Not apply	No	No	No	No	No	No	Low Quality	No	No	
**Osmonov et al. 2014** (49)	No	No	Yes	No	0	No	Yes	No	No	Yes	No	No	No	No	Low Quality	No	No	
**Passoni et al. 2014** (13)	No	Not apply	Not apply	Not apply	0	Not apply	Yes	Not apply	No	No	No	No	No	No	Low Quality	No	No	
**Suardi et al. 2015** (32)	Yes	Not apply	Not apply	Not apply	0	Not apply	Yes	Not apply	Yes	Yes	Yes	No	No	Yes	Acceptable	No	No	
**Claeyes et al. 2015** (53)	No	Not apply	Not apply	Not apply	0	Not apply	No	Not apply	No	No	No	No	Yes	No	Low Quality	No	No	
**Karnes et al. 2015** (9)	No	Not apply	Not apply	Not apply	0	Not apply	No	Not apply	No	Yes	No	No	No	Yes	Low Quality	No	No	
**Tilki et al. 2015** (33)	No	Not apply	Not apply	Not apply	0	Not apply	Yes	Not apply	No	Yes	Yes	No	Yes	Yes	Acceptable	No	No	
**Winter et al. 2015** (15)	Yes	Not apply	Not apply	Not apply	0	Not apply	No	No	No	Yes	No	No	No	No	Low Quality	No	No	
**Hijazi et al. 2015** (17)	No	Not apply	Not apply	Not apply	0	Not apply	No	Not apply	No	Yes	No	No	No	Yes	Low Quality	No	No	
**Osmonov et el. 2016** (50)	Yes	Not apply	Not apply	Not apply	16,7	No	Yes	Not apply	No	Yes	No	No	No	Yes	Low Quality	No	No	
**Zattoni et al. 2016** (35)	Yes	Not apply	Not apply	Not apply	0	Not apply	Yes	Not apply	No	Yes	Yes	No	No	Yes	Acceptable	No	No	
**Porres et al. 2017** (52)	No	Not apply	Not apply	Not apply	0	Not apply	Yes	Not apply	Yes	Yes	Yes	No	No	Yes	Acceptable	No	No	
**Montorsi et al. 2017** (54)	No	Not apply	Not apply	Not apply	0	Not apply	No	Not apply	No	Yes	No	No	No	No	Low Quality	No	No	
**Siriwardana et al. 2017** (20)	No	Not apply	Not apply	Not apply	0	Not apply	No	Not apply	No	Yes	No	No	Yes	No	Low Quality	No	No	
**Abreu et al. 2017** (55)	Yes	Not apply	Not apply	Not apply	0	Not apply	No	No	No	No	No	No	No	No	Low Quality	No	No	
**Herlemann et al. 2017** (21)	No	Not apply	Not apply	Not apply	0	Not apply	No	No	No	Yes	No	No	No	Yes	Low Quality	No	No	
**Otta-Oshiro et al. 2019** (36)	No	Not apply	Not apply	Not apply	0	Not apply	No	Not apply	No	No	No	No	No	No	Low Quality	No	No	
**Linxweiler et al. 2018** (56)	No	Not apply	Not apply	Not apply	0	Not apply	Yes	Not apply	No	No	No	No	No	No	Low Quality	No	No	
**Fossati et al. 2019** (57)	Yes	Not apply	Not apply	Not apply	186	No	Yes	Not apply	Yes	Yes	Yes	No	No	Yes	Acceptable	No	No	
**Hanske et al. 2019** (37)	yes	Not apply	Not apply	Not apply	0	Not apply	Yes	Not apply	No	Yes	No	No	Yes	No	Low Quality	No	No	

## DISCUSSION

Recent advances in imaging technology improved detection of isolated lymph node metastasis among some patients previously treated for PCa who recur. Typically, those patients were referred to long-term ADT. However, new concepts about oligometastasis as an intermediate state of tumor spread with a limited metastatic capacity has raised the possibility that salvage MDT may be effective to avoiding or delaying toxicity associated with use of systemic therapies (42, 43). Avoiding or delaying hormone therapy can improve patient's quality of life (44), however long-term functional and quality-of-life outcomes are not available yet. Despite that, literature have concern about prolonged exposure to ADT increases the risk of cardiovascular disease and diabetes in men aged >75 years diagnosed with PCa (45).

- Imaging methods to detect recurrence: Regarding modality of imaging methods to detect PCa nodal recurrence, some of the earliest reports of SLND investigated the use of PET/CT as a diagnostic tool. Schilling et al. (46) in 2008 reported histological findings among men with PCa and nodal recurrence determined by 11C-Choline PET/CT who underwent SLND and found positive histology for PCa in 7 of 10 cases. However, no information about PSA response was reported. Rinnab et al. (47) and Winter et al. (48) were the first to report the outcomes of SLND, with a complete response rate of 6.7% and 50%, respectively. Winter et al. (48) presented the data of six patients with BR after RP and positive solitary lymph node oligometastasis on 11C-choline PET/CT. All patients underwent SLND, and suspected areas were positive on pathology report. Following an average of 24 months, three patients had sustained PSA remission without any adjuvant therapy. Despite being small case series, those early results indicated that PSA remissions could be obtained after SLND in selected cases when PET/CT is used. Although 11C-Choline PET/CT, which has been more widely used in the diagnosis of relapse, has a high level of sensitivity (85.2%), its specificity is very low; specifically, the value was only 18.2% in the study by Osmonov et al. (49). In the study by Passoni et al. (13), 46 patients with BR of PCa after RP and a single positive lymph node finding were included. The objective of the study was to determine the positive predictive value (PPV) of 11C-Choline PET/CT or to identify the exact size and location of the lymph node with positive findings. The PPV appeared to be low, i.e., only 24%.
- Oncological outcomes: In a larger study by Rigatti et al. (31), 72 underwent SLND for nodal relapse (less than 3 sites on 11C-Choline PET/CT) after RP with complete PSA response rate of 57% after 40 days of SLND. At 5-years, cancer-recurrence free survival (CRFS) and biochemical-recurrence free survival (BRFS) were 34% and 19%, respectively. The CRFS was lower for patients with positive nodes in retroperitoneum versus solely in pelvis (11% vs. 53%). At a multivariable model using post-SLND data created by the authors, presence of pathologic nodes in the retroperitoneum, a higher number of positive lymph nodes and complete BR to SLND were independent predictors of clinical recurrence. The number and location of lymph nodes as predictors of clinical response highlight the importance of case selection for obtaining long-term results after SLND.
  In another large study, Osmonov et al. (50) evaluated cancer-specific survival (CSS) and overall survival (OS) in 54 patients with PCa recurrence who underwent extended SLND. The average follow-up was 43 months. Thirty-three patients (73.3%), achieved BCRF during follow-up. The mean BCRF-period was 32 months. CSS and OS were both 92% (3-year survival) and 80.6 (5-year survival), respectively.
  Another report by Jilg et al. (29) with 52 patients added adjuvant radiation therapy offered to all patients with positive nodes after SLND. Complete biochemical response after SLND was observed in 24/52 patients (46%). Twenty-seven of them (52%) received also adjuvant radiation therapy with mean dose of 50.8Gy. At 5-yrs, the CRFS and CSS were 25.6% and 77.7%, respectively. In this particular study, the receipt of radiation therapy renders the comparison with other pure SLND series difficult. In other study, Rischke et al. (51) showed among 93 patients, 46 patients had SLDN and 7 patients received radiotherapy in regions with proven lymph node metastases. Additional radiotherapy after SLDN resulted in delayed relapse within TR (5-year relapse-free rate 70.7 %) versus surgery only (5-year relapse-free rate 26.3%, <0.0001).
  Suardi et al. (32) reported the outcomes of 59 patients undergoing long-term SLND, with a 5-year observation period. They stated that the salvage procedure can be considered as a treatment option in patients with BR after RP and a positive finding on 11C-Choline PET/CT. Although most patients experienced another biochemical progression after the salvage therapy, nearly 40% of them experienced relapse-free survival.
  Tilki et al. (33) reported the outcomes of a cohort of 58 patients following SLND. The mean observation period following the surgery was 39 months. Thirteen patients (22.4%) experienced biochemical regression. Only one patient remained free of BR during the observation period. Clinical relapse was observed in 25 patients (48.1%) following surgery. Six patients (10.3%) died of PCa; four of them had extra-lymphatic preoperative findings on PET/CT. The authors reported that half of the patients had no clinical relapse, despite the occurrence of BR during the observation period; this may lead to the conclusion that, in some patients, salvage surgery may delay or even eliminate the onset of hormone therapy.
  Other studies used preoperative 68Ga-PSMA PET/CT. In the study by Porres et al. (52), 95 patients with rising PSA and nodal recurrence in 68Ga-PSMA PET/CT scan underwent SLND. Of this surgical cohort, 58% additionally underwent adjuvant/salvage radiation therapy (RT) and 18% received ADT before sLND. Complete BR was observed in 27.5% of patients and incomplete BR in 40.6%. In total, 62.2% of patients remained without ADT at follow-up. With a median follow-up of 21 months (1-75 months), the cancer-specific mortality rate was 3.7%. This study illustrates the multitude of neoadjuvant and adjuvant treatments that patients receive in the setting of nodal oligometastasis after primary treatment for PCa.
- Feasibility and Morbidityz: In our review, the SLND had mean duration ranging from 90 to 288 minutes and median blood loss was <250mL in all studies considered. Hospital stay ranged from 1 to 5d. Complications were mostly reported according to the Dindo-Clavien classification. Among complications, lymphorrhea, some cases of fever and wound complications were the most frequent. Most complications were of low grade. The rate of grade I complications ranged from 0% to 30%, grade 2 ranged from 0% to 25%, grade 3 and 4 ranged from 0% to 14% in all studies. Lymphocele drainage were the most frequent high-grade complication. Claeys et al. (53) showed among seventeen patients underwent open or minimally invasive salvage SLND, Clavien-Dindo grade 1, 2, 3a, and 3b complications were seen in 6, 1, 1, and 2 patients, respectively. They concluded SLND is feasible, but postoperative complication rate seems higher than that for primary LND. The minimally invasive approach using robot-assisted laparoscopy has been suggested to decrease surgical morbidity. Montorsi et al. (54) presented a feasible and effective procedure with acceptable short-term oncological outcomes. In another study about robotic-assisted approach, Abreu et al. (55) showed the minimally approach duplicated open surgery with superior nodal counts and decreased morbidity even expanding the template to retroperitoneum. Linxweiler et al. (56) showed safety and oncological effectiveness of robotic SLND in 36 patients with no high-grade complications occurred. They found robotic SLND is a feasible therapeutic option with low morbidity and can at least delay the initiation of ADT therapy.
  In another interesting study, Fossati et al. (57) reported the largest series of patients treated with SLND. They proposed that the oncological benefit may be limited to specific groups of patients. They tried to identify the optimal candidates for SLND based on preoperative characteristics. The study included 654 patients with nodal recurrence after RP and underwent SLND at nine tertiary referral centers. The imaging methods were PET/CT scan using either ^11^C-choline or ^68^Ga-labeled PSMA. At multivariable analysis, Gleason grade group 5, time from RP to PSA rising, ADT at PSA rising after RP, retroperitoneal uptake at PET/CT scan, three or more positive spots at PET/CT scan, and PSA level at SLND were significant predictors of clinical recurrence after SLND. This study is very attractive because recognized the optimal candidate to SLND based on routinely available preoperative characteristics despite any imaging-guided approach.
- Heterogeneity of studies: Given the heterogeneity of studies for SLND for early nodal recurrence after primary treatment for PCa, Ploussard et al. (38) recently performed a systematic review including 27 SLND series. The technique for detection of nodal recurrence among the selected studies was either choline or PSMA PET/CT. Overall, studies reported a relatively low morbidity (<10% of grade 3 or more by Clavien-Dindo scale) and a wide range of complete PSA response after SLND (13-79.5%). Mean follow-up was 29.4 months and 2- and 5-yr BRFS ranged from 23% to 64% and from 6% to 31%, respectively. Overall survival at 5-years was approximately 84%, however this review was very heterogeneous and it is hard to draw conclusions. This review included patients who had undergone radical prostatectomy as primary treatment, however, also included patients who received other modalities, such as RT, brachytherapy, or high-intensity focused ultrasound (HIFU) as primary treatment. We tried to perform a review with similar papers as possible. Accordingly, our study showed complete PSA responses with a wide range of 0 to >80% and a relatively low morbidity, with grade 3 complications varying in the range of 1.69-30%.
- Review method: We used for our methodological quality analysis the SIGN Checklist protocol, which is based on strict quality criteria and our evaluation classified most of the articles as non-eligible in methodological quality, since Ploussard et al. (38) review was based on another methodology, the modified Delphi technique, with criteria simpler for accepting series of cases. However, the conclusions of both studies followed the same lines, that is, we still do not have the quality studies to base ourselves and make the best conclusions. What we have evaluated in our study, so far, that patients undergoing SLND most delay the use of ADT, but few remain without BR for many years.
- Limitations: The selected studies in our analysis had several limitations; our methodological analysis rated only eight articles as acceptable, and no high-quality studies found. This is mainly because all but one were cohort studies, the exception was two case-control studies. Randomized clinical trials are lacking. Considering the marked heterogeneity in the studies, a meta-analysis cannot be performed in this setting.

## CONCLUSIONS

Although evidence from clinical trials are lacking to date, MDT for relapsing oligometastatic nodal PCa using SLND seems to be a safe and effective treatment for cancer control in selected patients. The long-term impact in overall survival and quality of life, as well as.
